# C-Phycocyanin–Cisplatin Combination Targeting Redox Balance for Enhanced Efficacy Against Glioblastoma Cells

**DOI:** 10.32604/or.2025.070729

**Published:** 2025-11-27

**Authors:** Rym Akrout, Ludovic Leloup, Khouloud Ayed, Fabrice Parat, Sami Zekri, Wassim Y. Almawi, Rahma Boughriba, Hanen Attia, Olfa Masmoudi-Kouki, Hervé Kovacic, Asma Gati

**Affiliations:** 1 Laboratory of Genetics Immunology and Human Pathology, Biology Department, Faculty of Sciences of Tunis, University of Tunis El Manar, Tunis, 2092, Tunisia; 2 Faculté des Sciences Médicales et Paramédicales, Institut de NeuroPhysiopathologie (INP), UMR 7051, CNRS, Aix Marseille Université, Marseille, 13005, France; 3 Confocal Microscopy Unit, Faculty of Medicine of Tunis, University Tunis El Manar, Tunis, 1007, Tunisia; 4 Laboratory of Neurophysiology, Cellular Physiopathology and Biomolecules Valorisation, LR18ES03, Faculty of Sciences of Tunis, University of Tunis El Manar, Tunis, 2092, Tunisia

**Keywords:** Chemosensitivity, cisplatin, C-phycocyanin, glioblastoma, redox-targeted therapy

## Abstract

**Objectives:**

Cisplatin (CDDP) therapy for glioblastoma (GBM) is linked with several limitations, which include poor penetration of the blood-brain barrier (BBB), systemic toxicity, and the development of drug resistance mechanisms implicating oxidative stress dysregulation and compromised apoptotic pathways. This study evaluates C-Phycocyanin (C-PC) as a potential adjuvant to enhance CDDP efficacy by modulating redox balance and apoptosis.

**Methods:**

GBM cells (U87 and U87-EGFRvIII) were treated with CDDP, C-PC, or their combination. Cell viability was assessed by MTT assay; apoptosis was evaluated by DAPI staining and Western blot analysis of cleaved Caspase-3 and poly (ADP-ribose) polymerase (PARP). Both intracellular and extracellular reactive oxygen species (ROS) were measured using 2^′^,7^′^-dichlorodihydrofluorescein diacetate (DCF-DA) fluorescence and lucigenin chemiluminescence, respectively. Catalase activity was quantified via hydrogen peroxide (H_2_O_2_) decomposition assay, and manganese superoxide dismutase (MnSOD) expression by Western blot.

**Results:**

C-PC selectively decreased U87 GBM cell viability while sparing normal cells. C-PC enhanced CDDP cytotoxicity, reducing viability to 26.5% vs. 53.2% for CDDP alone. This effect correlated with increased apoptosis, evidenced by DNA fragmentation and higher cleaved caspase-3 and PARP levels. Combined treatment lowered ROS below survival thresholds while upregulating MnSOD and catalase activity. In U87-EGFRvIII cells, CDDP reduced viability modestly (85.2%), C-PC alone decreased viability significantly (51.5%) and induced cell death, but the combination did not further increase apoptosis. Here, C-PC’s pro-apoptotic effects, alone or with CDDP, were also associated with reduced oxidative stress in cells.

**Conclusion:**

We demonstrate that C-PC enhances CDDP cytotoxicity in sensitive U87 cells by promoting apoptosis and modulating ROS, suggesting potential for improved therapeutic efficacy with reduced systemic toxicity. Compared to the combination, C-PC monotherapy achieves superior cytotoxicity in CDDP-resistant U87-EGFRvIII cells, underscoring its potential as a standalone therapeutic approach for chemotherapy-resistant glioblastoma subtypes.

## 1 Introduction

Glioblastoma (GBM) is the most common and lethal primary brain tumor in adults [1]. It is characterized by rapid cellular proliferation, marked invasiveness, and resistance to conventional treatment modalities. It is associated with poor prognosis and a low median survival time of 12 to 15 months post-diagnosis [1,2]. This poor outcome is reportedly attributed to several factors, including limited drug delivery across the blood-brain barrier (BBB), dose-limiting systemic toxicity, and resistance to drug treatment, including cisplatin (CDDP) [2,3]. The clinical application of CDDP in GBM treatment is particularly constrained by its limited BBB penetration, resulting in subtherapeutic intratumoral exposure [4]. Furthermore, Severe dose-limiting toxicities, including nephrotoxicity, peripheral neuropathy, ototoxicity, and myelosuppression, necessitate dose reductions or even discontinuation [2,5]. In addition, the efficacy of CDDP is compromised by both intrinsic and acquired resistance mechanisms, notably metabolic reprogramming that allows tumor cells to adapt to oxidative stress, thereby sustaining tumorigenesis and chemoresistance [6,7].

Cancer cells display a higher metabolic rate and elevated baseline levels of reactive oxygen species (ROS) compared to normal cells [3]. ROS play a dual role in cancer progression [8]. Moderate ROS levels support the survival of malignant cells by enabling evasion of cell death and contribute to resistance to chemotherapy [9], but compromise cancer cell survival below a critical ROS threshold [10]. As such, maintaining redox balance is critical for cancer cell survival [8,9]. ROS levels are controlled by glutathione peroxidase (GPx), catalase, superoxide dismutase (SOD), and other antioxidant enzymes [11]. The mammalian SOD family comprises the cytosolic Cu/Zn-SOD (SOD1), mitochondrial Mn-SOD (SOD2), and the extracellular (Ec)-SOD (SOD3) [11]. These act by converting the superoxide (O_2_·^−^) to hydrogen peroxide (H_2_O_2_), which is subsequently detoxified to water by catalase and GPx [12]. It was shown that Mn-SOD acts as a tumor suppressor [13], and its downregulation in cancers, including GBM, was linked with enhanced cell proliferation and survival, while its overexpression inhibits the growth of cancer cells and promotes apoptosis in GBM [14].

Antioxidant therapies, based on lowering ROS levels in cancer cells, hold promise for treating tumors and overcoming drug resistance [15]. The DNA cross-linking anti-cancer drug, CDDP, exerts its effects by inducing apoptosis and downregulating ROS production [16]. GBM cells reportedly develop treatment resistance through various mechanisms, including enhanced antioxidant defenses, compromised DNA repair pathways, and inhibition of apoptosis [17,18]. Accordingly, targeting these resistance mechanisms through combination therapies could improve the therapeutic efficacy of CDDP [18]. Recent studies reported the therapeutic capacity of natural compounds, including C-Phycocyanin (C-PC), as adjuvants to conventional chemotherapy [19,20]. A significant pigment in the blue-green algae *Spirulina*, C-PC, reportedly possesses potent antioxidant, anti-inflammatory, and anti-cancer activities [20,21]. These were attributed to their capacity to inhibit cell cycle progression, induce apoptosis, promote autophagy, and improve the therapeutic efficacy of various chemotherapeutic agents [21]. While this presents an attractive strategy for overcoming CDDP resistance, the potential role of C-PC in GBM treatment remains mainly unexplored [22].

In this study, we investigate the combined effects of C-PC and CDDP on GBM cell lines, focusing on the induction of apoptosis and modulation of ROS. We will test the notion that C-PC monotherapy is an effective treatment modality in CDDP-resistant GBM cells, thereby promoting its inclusion as an alternative approach to improve GBM treatment outcomes and overcome chemoresistance. As a proof-of-concept study, we hypothesize that C-PC augments CDDP-induced cytotoxicity by modulating oxidative stress and facilitating apoptotic pathways. While CDDP is not the clinical standard for GBM treatment, understanding these mechanistic interactions may inform future combination strategies with clinically relevant agents.

## 2 Materials and Methods

### 2.1 Cell Lines and Culture Conditions

The U87 human GBM cell line (ATCC #HTB-14) was obtained from the American Type Culture Collection (ATCC; Manassas, VA, USA). The U87-EGFRvIII cell line, engineered to overexpress the epidermal growth factor receptor variant III (EGFRvIII) in U87 cells, was generously provided by Dr. Eddy Pasquier from the Cancer Research Center of Marseille (CNRS, UMR7258, Marseille, France). Human Umbilical Vein Endothelial Cells (HUVEC) were kindly provided by Prof. Najet Srairi-Abid from the Institut Pasteur de Tunis (LR20IPT01, Tunis, Tunisia). All cell types were cultured by regular passages in media supplemented with 10% fetal bovine serum (Gibco, Waltham, MA, USA) at 37°C in a humidified atmosphere containing 5% CO_2_. HUVEC cells were cultured in RPMI-1640 media (Sigma-Aldrich; St. Louis, MO, USA) while U87 and U87-EGFRvIII cell lines were maintained in EMEM medium (Lonza, Levallois Perret, France). All cell lines were routinely tested for mycoplasma contamination using DAPI staining (Abcam, Cambridge, UK) and authenticated by Short Tandem Repeat (STR) profiling.

### 2.2 Cell Viability Assay

Cell viability was evaluated using the 3-(4,5-dimethylthiazol-2-yl)-2,5-diphenyltetrazolium bromide (MTT; Sigma-Aldrich, Darmstadt, Germany) assay. Cells were seeded in 96-well plates at a density of 7000 cells/well in triplicate and incubated overnight to allow attachment. Cells were treated for 24 h with C-PC (HS code: 12122100; Bio Algues, Mahdia, Tunisia) at 5, 25, 50, or 100 μg/mL, and/or CDDP (Cytopharma Oncology, Zaghouan, Tunisia) (Medical Authorization Number: 9383071H) at 5, 10, or 15 μM. The C-PC range used (5–100 μg/mL) reflects reported anticancer activity, with 100 μg/mL approaching the upper limit before evident toxicity in normal cells. The CDDP concentrations were selected based on ranges commonly used *in vitro* to assess cytotoxic responses; 10 μM was used in combination assays to provide moderate cytotoxicity suitable for subsequent combination testing. Following treatment, the medium was replaced with fresh medium containing 0.5 mg/mL of MTT solution, and the plates were incubated for an additional three hours at 37°C. Formazan crystals produced by viable cells were dissolved using DMSO (Sigma-Aldrich, Darmstadt, Germany), and Absorbance was measured at 560 nm using a Multiskan RC plate reader (Thermo-Labsystems, Waltham, MA, USA). Cell viability was calculated using the formula: Cell Viability (%) = (OD of Treatment/OD of Control) * 100. All experiments were done in triplicate, and results represent the mean of three independently performed assays.

### 2.3 C-PC Cellular Uptake and Morphological Analysis

C-PC cellular uptake and morphological changes were evaluated in U87 and U87-EGFRvIII GBM cell lines. For C-PC uptake analysis, cells were treated with 100 μg/mL of C-PC at a density of 2 × 10^5^ cells/well for 24 h at 37°C in 6-well plates. Untreated cells were used as negative controls to account for autofluorescence. Cells were trypsinized using 0.25% trypsin-EDTA (1X; Gibco) after treatment, washed twice with phosphate-buffered saline (PBS, 1X, pH 7.4; Gibco), and analyzed by flow cytometry using a fluorescence-activated cell sorting (FACS) Canto II (Becton Dickinson, San Jose, CA, USA). Uptake was quantified based on the mean fluorescence intensity (MFI) of C-PC. Gating strategy included exclusion of debris and doublets, and analysis was restricted to the main population of viable cells. For morphological analysis, U87 and U87-EGFRvIII cells were seeded at 6 × 10^4^ cells/well in 24-well plates and incubated overnight. Cultured cells were treated with 100 μg/mL of C-PC, 10 μM of CDDP, or C-PC-CDDP combination for 24 h under standard culture conditions. Morphological changes were observed and documented using a phase-contrast inverted microscope (Leica DMI3000B, Wetzlar, Germany).

### 2.4 Nuclear Staining via DAPI

The U87 and U87-EGFRvIII GBM cell lines were seeded on poly-L-lysine (Sigma-Aldrich) -coated coverslips in a 6-well plate (1 × 10^5^ cells/well), treated for 24 h at 37°C with C-PC, CDDP, or their combination, and incubated overnight. Cells were washed with PBS (1X, pH 7.4) and fixed with 4% formaldehyde for 20 min at room temperature (RT). Cells were stained using Mounting Medium With DAPI-Aqueous, Fluoroshield (Abcam, Cambridge, UK), which contains DAPI at a final concentration of 1.5 μg/mL (equivalent to 0.0002% w/v). One drop of the mounting medium was added to each coverslip, which was then gently covered with a slide to prevent air bubbles. Samples were visualized and photographed using a Zeiss Axio Observer 7 Apotome III confocal microscope (Zeiss, Jena, Germany) (at 200× magnification). DNA condensation was analyzed using ImageJ software (version 1.54 g, National Institutes of Health (NIH), Bethesda, MD, USA) to assess nuclear morphology and detect apoptotic changes.

### 2.5 Western Blot Analysis

The U87 and U87-EGFRvIII cell lines were treated for 24 h, followed by lysis with a freshly prepared Radio-Immunoprecipitation Assay (RIPA) buffer (#89900; Thermo Scientific, Waltham, MA, USA) for 30 min at 4°C with agitation. The cell lysates were centrifuged at 10,000× *g* for 10 min at 4°C, and the total protein concentration in each sample was measured in triplicate using the Pierce™ Bradford Plus Protein Assay Kit (#89900; Thermo Scientific). Samples were normalized to ensure equal loading of 35 μg of total protein from samples onto 10% or 12% SDS-polyacrylamide gel (PAGE). To ensure accurate quantitative comparisons, GAPDH, a housekeeping protein, was used as a loading control to confirm equal protein loading and transfer efficiency. After separation, proteins were transferred to a nitrocellulose membrane (Amersham Protan, GE Healthcare, Chicago, IL, USA), which was blocked with 5% non-fat dry milk in TBST (1× TBS plus 0.05% Tween 20; Sigma-Aldrich, Saint-Quentin-Fallavier, France) for one hour at RT and incubated overnight at 4°C with specific primary antibodies.

The membrane was then washed three times with TBST (Tris-buffered saline + Tween-20) and incubated with horseradish peroxidase (HRP)-conjugated secondary antibodies for one hour at RT. Following a 3× wash with TBST, the membrane was treated with chemiluminescent HRP substrate (Millipore, Darmstadt, Germany) for one minute. Protein bands were detected using a Syngene G-box imaging system (Syngene, Cambridge, UK), and band intensities were visualized using ImageJ software (NIH) (version 1.54 g). The following primary antibodies were used at 1:1000 dilution: anti-pro Caspase-3 (#ab32499; Abcam, Cambridge, UK), anti-Caspase 3 (#9662S; Cell Signaling Technology, Danvers, MA, USA), anti-PARP (#9542; Cell Signaling Technology), anti-MnSOD (#13141S; Cell Signaling Technology), and anti-α-Tubulin (#2144; Cell Signaling Technology), while anti-GAPDH (#G8795; Sigma Aldrich) was used at 1:20,000 dilution. HRP-conjugated secondary antibodies, horse anti-mouse IgG (#7076; Cell Signaling Technology) and goat anti-rabbit IgG (#7074; Cell Signaling Technology), were both used at 1:1000 dilution.

### 2.6 Measurement of Extracellular superoxide (O_***2***_·^−^)

NADPH oxidase activity was determined by measuring extracellular O_2_·^−^ production in U87 and U87-EGFRvIII GBM cell lines. Cells were seeded in 96-well white plates at a density of 20,000 cells/well and incubated overnight. Following treatment with C-PC, CDDP, or their combination for one hour, the supernatant was removed, and cells were incubated in phenol red-free culture media containing 30 μM of lucigenin (Sigma-Aldrich) and 1 mM of β-Nicotinamide adenine dinucleotide phosphate (NADPH; Thermo Fisher Scientific, Waltham, MA, USA) (the cofactor for NADPH oxidases). O_2_·^−^ generation was monitored by measuring luminescence values every minute for one hour at 37°C, using a Fluoroskan plate reader (Fluoroskan Ascent FL, Thermofischer, Vantaa, Finland). Data obtained was expressed as a percentage of O_2_·^−^ production relative to untreated cells.

### 2.7 Measurement of Intracellular Hydrogen Peroxide (H_***2***_O_***2***_)

Intracellular ROS production was measured using dichlorodihydrofluorescein diacetate (H_2_-DCFDA; Invitrogen, Waltham, MA), a fluorescent probe sensitive to ROS. In brief, U87 and U87-EGFRvIII cells were seeded at 20,000 cells per well in black 96-well plates and incubated overnight for attachment. Cells were treated with C-PC, CDDP, or their combination at the indicated concentrations for one hour. The medium was removed following treatment, and the cells were incubated for 30 min at 37°C in a buffer containing 10 μM of H_2_-DCFDA. Following incubation, the cells were washed, and fluorescence was detected using the Fluoroskan Ascent FL fluorimeter (Thermofischer Scientific, Vantaa, Finland) at an excitation/emission wavelength of 490/538 nm. Results were expressed as the percentage variation relative to control cells.

### 2.8 Catalase Activity Measurement

Catalase activity was determined by measuring the rate of hydrogen peroxide (H_2_O_2_) decomposition. The U87 and U87-EGFRvIII GBM cells were incubated in 25-mm flasks at 37°C for 24 h, with or without treatment. After incubation, the cells were washed twice with PBS (1X, pH 7.4), and total cellular proteins were extracted using a lysis buffer containing EDTA (10 mM), Tris–HCl (50 mM; pH 8; Sigma-Aldrich), Triton X-100 (1%; Sigma-Aldrich), and PMSF (100 μM; Sigma-Aldrich). The cell lysate was centrifuged at 16,000× *g* for 25 min at 4°C to obtain clear supernatants. Protein samples were mixed with 30 mM H_2_O_2_ in PBS, and the reduction in H_2_O_2_ was measured at 240 nm over 3 min using a UV-3100PC spectrophotometer (Avantor, Radnor, PA, USA). Catalase activity was determined using the extinction coefficient of 40/mM/cm for H_2_O_2_.

### 2.9 Statistical Analysis

Statistical analysis was performed using GraphPad Prism 8.0.1 software (GraphPad Software, San Diego, CA, USA), and all data were obtained from at least three independently performed experiments presented as the mean ± standard deviation (SD). Differences within a single cell line were analyzed using the Kruskal–Wallis test followed by Dunn’s post-hoc test for multiple comparisons, while comparisons between two groups were performed using the Mann–Whitney test. A *p*-value < 0.05 was considered statistically significant.

## 3 Results

### 3.1 C-PC Selectively Reduces GBM Cell Viability while Sparing Normal Cells

**Figure 1:**
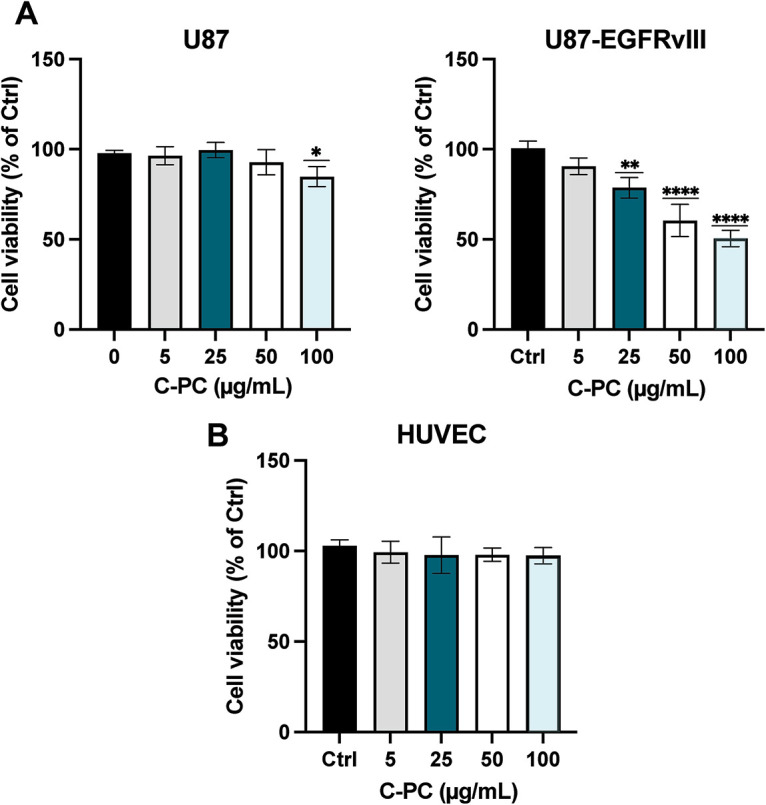
Effect of C-PC on the viability of (**A**) GBM cell lines (U87 and U87EGFRvIII) and (**B**) HUVEC cells were treated with varying concentrations of C-PC (0, 5, 25, 50, and 100 μg/mL) for 24 h, and cell viability was assessed by MTT assay. Data represent mean ± SD; from 3 independent experiments. The percentage of viability is presented relative to untreated cells. *p < 0.05, **p < 0.01, ****p < 0.0001

The cytotoxic effects of C-PC were evaluated in sensitive (U87) and resistant (U87-EGFRvIII) GBM cells, as well as in non-tumorigenic HUVEC cells. Cells were treated for 24 h with C-PC at concentrations ranging from 5 to 100 μg/mL, with untreated cells serving as controls. As shown in Fig. 1A, C-PC treatment resulted in higher cytotoxicity against U87-EGFRvIII cells compared to U87 cells. Treatment with 100 μg/mL of C-PC reduced cell viability by 51.19% in U87-EGFRvIII cells (*p* < 0.0001), while U87 cells showed a modest reduction of 10% (*p* = 0.04). As shown in Fig. 1B, C-PC exhibited no cytotoxicity towards normal HUVEC cells, highlighting its selective toxicity towards cancer cells.

### 3.2 Enhanced C-PC Uptake in U87-EGFRvIII Cells Compared to U87 Cells

**Figure 2:**
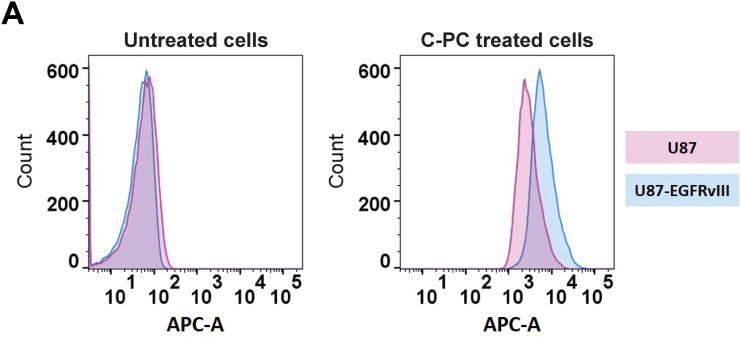

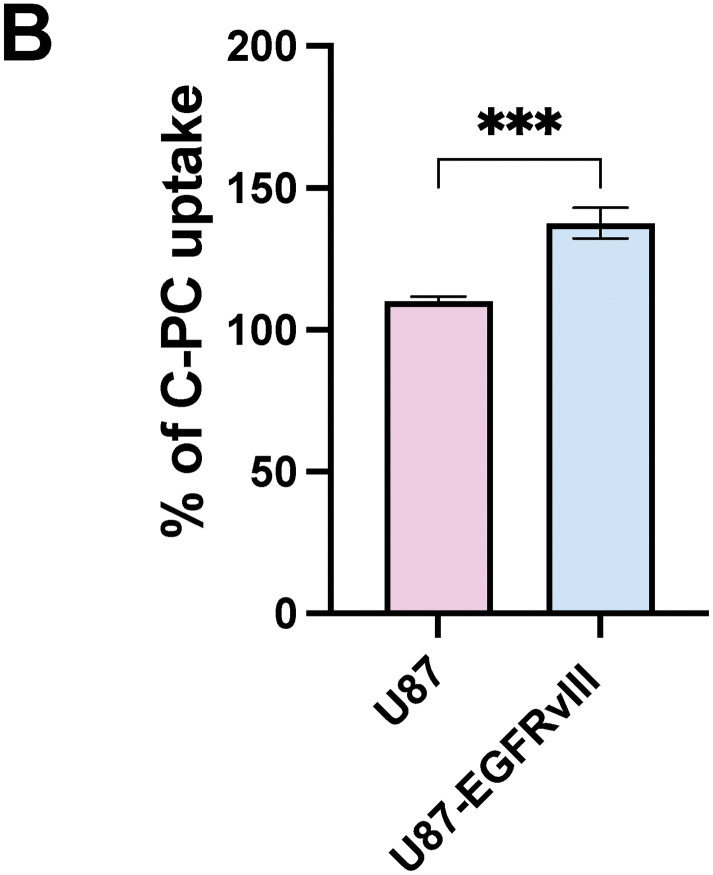
(**A**) Flow cytometry analysis of C-PC uptake by GBM cell lines after 24 h. (**B**) Quantitative analysis of the mean fluorescence intensity of C-PC in GBM cells. Results are expressed as the mean ± SD from 3 independently performed experiments. ***p < 0.001

The cellular uptake, and thus increased cytotoxicity, of C-PC in U87-EGFRvIII cells following a 24-h treatment period was evaluated using flow cytometry. The results showed a significantly higher red fluorescence shift in U87-EGFRvIII cells (134.3%) compared to U87 cells (111.1%), indicating a greater intracellular accumulation of C-PC in the (resistant) U87-EGFRvIII cell line (*p* < 0.001). This enhanced C-PC uptake in U87-EGFRvIII cells likely contributes to C-PC-mediated cytotoxicity (Fig. 2A,B), suggesting that this differential mechanism may play a role in the selective toxicity exerted by C-PC on resistant GBM cells.

### 3.3 C-PC and CDDP Exert Enhanced Cytotoxicity in U87 Cells, without Additional Toxicity in U87-EGFRvIII Cells

**Figure 3:**
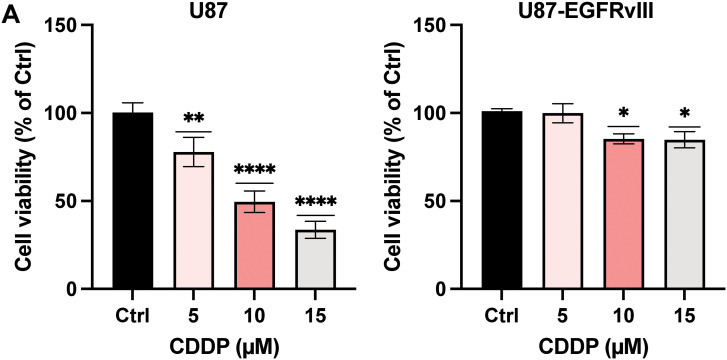

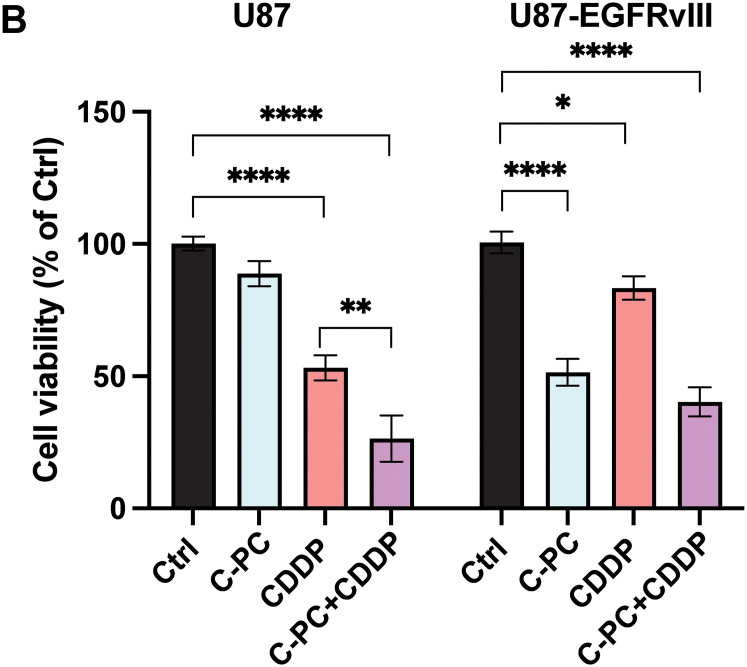
Effects of CDDP alone or in combination with C-PC on GBM cell viability. (**A**) U87 and U87EGFRvIII cells were treated with CDDP at the indicated concentrations for 24 h. Cell viability was measured using the MTT assay. (**B**) GBM cells were treated with 100 μg/mL C-PC and 10 μM CDDP, either individually or in combination, for 24 h. Cell viability was determined using the MTT assay. Results are presented as the mean ± SD of three individual experiments. *p < 0.05, **p < 0.01, ****p < 0.0001

The combined cytotoxic effects of C-PC and CDDP were evaluated using the MTT assay in U87 and U87-EGFRvIII GMB cells. CDDP (10 μM) exhibited high cytotoxicity in U87 cells, resulting in a 50% reduction in viability, compared to a 14.81% reduction in viability in U87-EGFRvIII cells (Fig. 3A). This confirms the resistance of U87-EGFRvIII cells to CDDP. Subsequently, a C-PC concentration of 100 μg/mL, alongside 10 μM CDDP, was selected for combined treatment over 24 h. This combination therapy significantly decreased the cell viability of U87 cells to 26.46%, compared to 51.69% with CDDP and 88.78% with C-PC monotherapy (*p* < 0.0001) (Fig. 3B). This indicates an additional effect resulting from a non-cytotoxic C-PC concentration combined with a low CDDP concentration, potentially minimizing side effects on normal cells. Conversely, in U87-EGFRvIII, the combination treatment did not alter cytotoxicity, with viability reduced to 51.49% compared to C-PC alone (48.06%) (Fig. 3B). This indicates that while C-PC alone effectively targets resistant U87-EGFRvIII cells, it does not overcome CDDP resistance. However, C-PC monotherapy is more effective against CDDP-resistant GBM cells, while its combination with CDDP confers enhanced efficacy in sensitive U87 cells.

### 3.4 C-PC and CDDP Induce Cellular Morphological Changes and DNA Fragmentation in GBM Cells

**Figure 4:**
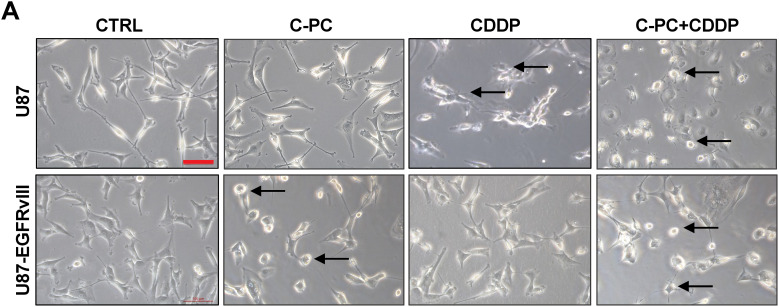

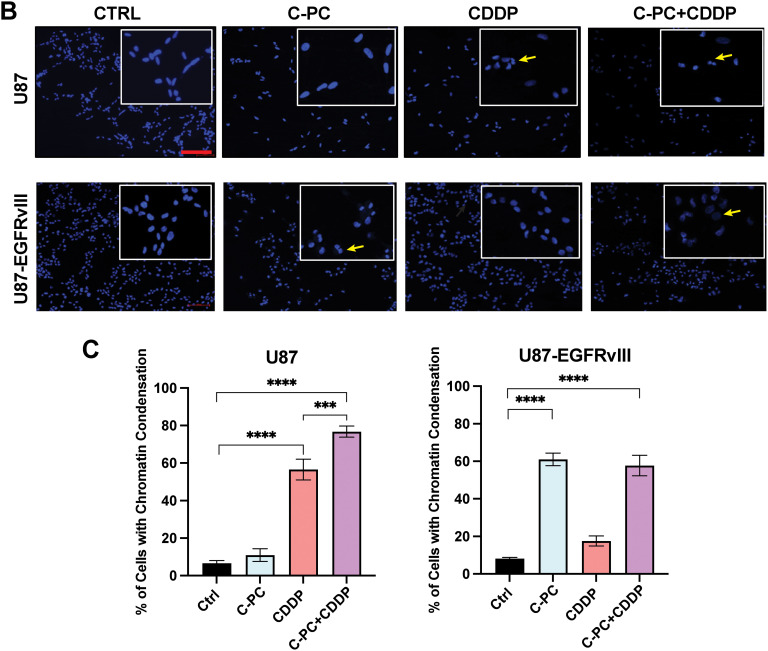
Effects of C-PC alone or in combination with CDDP on GBM cells and nuclear morphology. (**A**) Cell morphological changes in U87 and U87EGFRvIII cells after 24-h treatment with C-PC (100 μg/mL) and CDDP (10 μM), alone or in combination, were observed under phase-contrast microscopy. Black arrows point to cells exhibiting morphological changes, including shrinkage, rounding, and detachment. Scale bar: 100 μm; objective: 20×. (**B**) Nuclear morphology assessment of GBM cells by DAPI staining using confocal microscopy. Yellow arrows point to DNA condensation and fragmentation. Scale bar: 100 μm; objective: 200×. (**C**) Quantification of DNA-condensed cells as a percentage of total nuclei. Data are shown as mean ± SD. ***p < 0.001, ****p < 0.0001 vs. untreated cells

To evaluate the cytotoxic mechanisms of C-PC individually and in combination with CDDP, we examined the morphology of GBM cells using light microscopy (Fig. 4A) and the nuclear morphology via DAPI staining (Fig. 4B). Treating U87 cells with CDDP alone induced structural changes, including cell shrinkage, rounding, and detachment. Co-treatment with CDDP and C-PC significantly intensified these effects, resulting in increased DNA fragmentation (75.47%), compared to CDDP alone (58.00%) (Fig. 4C). In contrast, U87-EGFRvIII cells exhibited minimal morphological changes following CDDP treatment, with a low percentage of apoptotic nuclei detected by DAPI staining (17.53%). Furthermore, combining C-PC and CDDP did not enhance cytotoxicity compared to C-PC alone, with similar DNA fragmentation levels (57.7%) as those observed with C-PC alone (60.89%) (Fig. 4C). This indicates that C-PC effectively induces morphological changes and DNA fragmentation in U87 cells when combined with CDDP. As U87-EGFRvIII cells remain resistant to CDDP, C-PC alone is the more effective treatment for this resistant cell line.

### 3.5 C-PC and CDDP Trigger Apoptosis via Caspase-3 and PARP Activation in GBM Cells

**Figure 5:**
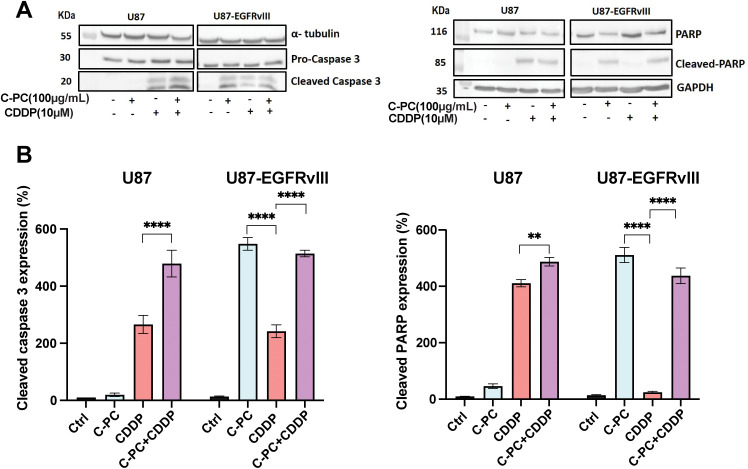
Effects of C-PC alone or in combination with CDDP on apoptosis in GBM cell lines. (**A**) GBM cells were treated with the indicated concentrations and treatments for 24 h. Cleaved caspase-3 and cleaved PARP fragments were assayed by Western blotting; representative data are shown from three individual experiments (*n* = 3). Full-length original blots are presented in Fig. A1. (**B**) Data from Western blot analysis in (**A**) were quantified using ImageJ software. **p < 0.01, ****p < 0.0001

To evaluate the mechanism of cell death induced by C-PC and CDDP, we assessed the expression levels of key apoptosis mediators, cleaved caspase-3 and PARP, by Western blot (Fig. 5A). Co-treatment of U87 cells with C-PC and CDDP resulted in significantly increased caspase-3 and PARP activation compared to either agent alone (Fig. 5B), indicating an enhanced apoptotic effect. In contrast, C-PC treatment alone effectively induced caspase-3 and PARP cleavage in U87-EGFRvIII cells (*p* < 0.0001), whereas the combination with CDDP did not further enhance apoptosis. This indicates that C-PC alone can activate apoptotic pathways in the resistant U87-EGFRvIII cells (Fig. 5B).

### 3.6 C-PC Alone or Combined with CDDP Reduces ROS Production in GBM Cells

**Figure 6:**
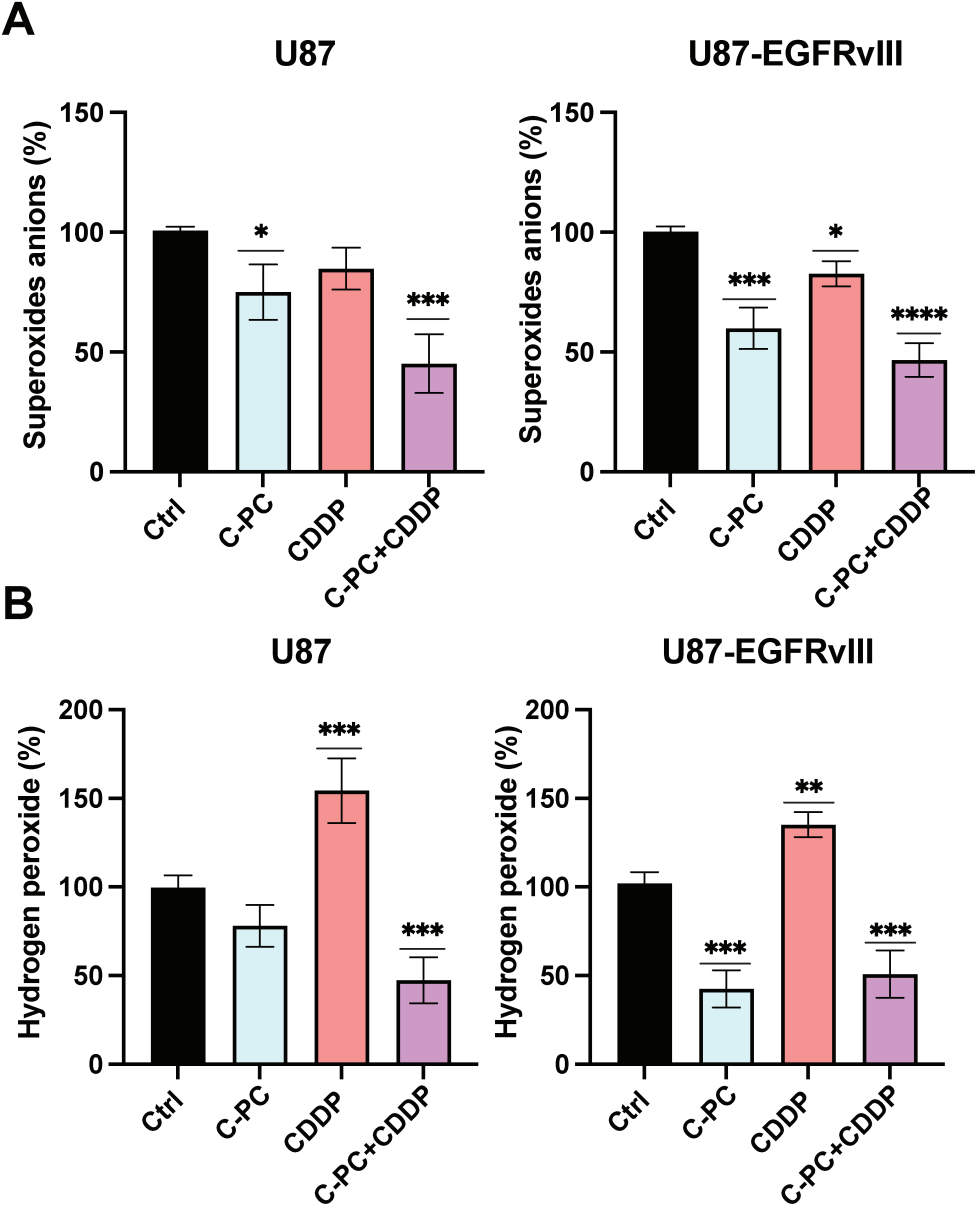
Production of O_2_·^−^ and H_2_O_2_ in GBM cell lines. U87 and U87EGFRvIII were treated with 100 μg/mL C-PC alone or in combination with 10 μM CDDP for 1 h. (**A**) O_2_·^−^ production was measured using lucigenin-enhanced chemiluminescence. (**B**) H_2_O_2_ generation was determined using the DCFDA assay. Data from three different experiments are presented as mean ± SD. *p < 0.05, **p < 0.01, ***p < 0.001, ****p < 0.0001 compared to controls

We evaluated the effects of C-PC, CDDP, and their combination on ROS generation in GBM cells by measuring extracellular O_2_·^−^ and intracellular H_2_O_2_ levels in U87 and U87-EGFRvIII cells. Extracellular O_2_·^−^ levels were quantified by the lucigenin chemiluminescence assay, with untreated cells normalized to 100%. Following one hour of treatment, the combination of C-PC and CDDP significantly reduced O_2_·^−^ production in U87 cells by 54.77% (*p* < 0.001; Fig. 6A), compared to 24.95% reduction with C-PC alone (*p* < 0.05; Fig. 6A). In U87-EGFRvIII cells, C-PC alone and in combination with CDDP reduced O_2_·^−^ production by 40.07% and 53.3%, respectively (*p* < 0.001, *p* < 0.0001; Fig. 6A).

Intracellular H_2_O_2_ levels were measured using the DCFDA assay under the same conditions. CDDP treatment alone increased H_2_O_2_ production by 170% in (sensitive) U87 cells and 130% in resistant U87-EGFRvIII cells. In contrast, C-PC treatment reduced H_2_O_2_ levels in U87-EGFRvIII cells, with no significant changes observed in U87 cells. The C-PC and CDDP combination resulted in a significant decrease in H_2_O_2_ production in U87 (52.63%) and U87-EGFRvIII (49.07%) cells (Fig. 6B).

Taken together, this suggests that C-PC, either alone or in combination with CDDP, reduces ROS production in GBM cells. Depending on the cell type, this reduction in H_2_O_2_ and O_2_·^−^ production contributes to reduced cell viability and enhanced cell death. C-PC appears to reduce ROS levels below the threshold needed for cancer cell survival by suppressing ROS production or enhancing their clearance through antioxidant mechanisms.

### 3.7 C-PC Alone or Combined with CDDP Upregulates MnSOD Expression and Catalase Activity in GBM Cells

**Figure 7:**
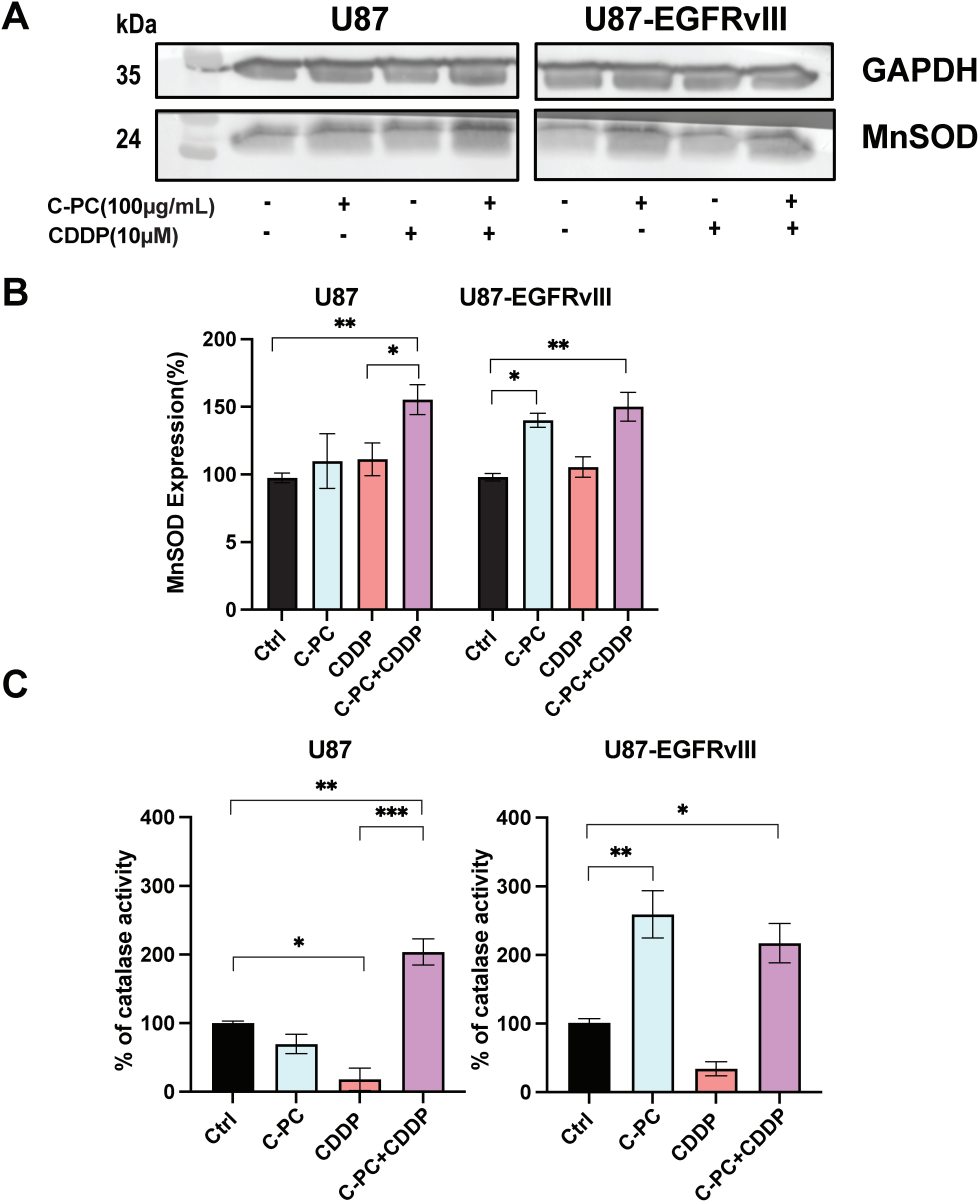
Effect of C-PC and CDDP treatment on MnSOD expression and catalase activity in GBM cells. U87 and U87EGFRvIII cells were treated for 24 h with the indicated treatments. (**A**) MnSOD expression was assessed by Western blotting and quantified using ImageJ software. Full-length original blots are presented in Fig. A2. (**B**) Data from Western blot analysis in (**A**) were presented as mean ± SD from three independent assays. *p < 0.05, **p < 0.01, compared with untreated cells. (**C**) Catalase activity was measured based on the rate of H_2_O_2_ decomposition. Results are expressed as mean ± SD from three independently performed experiments; catalase activity is presented as a percent of untreated cells. *p < 0.05, **p < 0.01, ***p < 0.001

As MnSOD and catalase are key antioxidants in glioma progression and cellular defences against oxidative stress, we evaluated their expression in U87 and U87-EGFRvIII cells. Western blot analysis revealed that neither C-PC nor CDDP alone significantly affected MnSOD levels; however, the combination of C-PC and CDDP significantly increased MnSOD expression in U87 cells by 155.85% compared to untreated controls. In comparison, C-PC alone increased MnSOD expression by 144.14%, and to 155.09% when combined with CDDP in U87-EGFRvIII cells (Fig. 7A,B). Similarly, co-treatment with C-PC and CDDP significantly increased catalase activity in U87 cells to 203.81%. In U87-EGFRvIII cells, C-PC alone augmented catalase activity to 259.19%, and 217.07% when combined with CDDP (Fig. 7C). These findings indicate that C-PC, alone and in combination with CDDP, upregulates MnSOD expression and enhances catalase activity, thereby reducing the cells’ ability to scavenge O_2_·^−^ radicals and lowering ROS levels, which in turn reduces overall cellular oxidative stress.

## 4 Discussion

Characterized by rapid progression, resistance to therapy, and poor prognosis, GBM remains the most aggressive and lethal form of adult brain tumor [2]. Despite its widespread use, the treatment efficacy of CDDP is often limited by the ability of the tumor to manage oxidative stress and evade apoptosis, highlighting the need for alternative treatment options. In this context, pharmacologically active marine-derived compounds were evaluated as potential chemotherapy [23], of which C-PC, a biliprotein extracted from the cyanobacteria *Arthrospira platensis*, has demonstrated potent anticancer effects in various malignancies [24], but not extensively for GBM. Our study evaluated the therapeutic potential of C-PC either as a standalone agent or in combination with CDDP in U87 and U87-EGFRvIII cells, particularly by targeting redox homeostasis and enhancing apoptosis.

Key conclusions regarding the therapeutic potential of C-PC in GBM emerge from our study. C-PC is selectively cytotoxic against GBM cells, while sparing normal cells. The C-PC-CDDP combination therapy exhibits an additive effect in chemotherapy-sensitive cells, mediated by enhanced apoptosis and redox modulation. Furthermore, C-PC monotherapy represents a viable treatment option for chemotherapy-resistant GBM subtypes, thus tackling a critical clinical need. It is worth acknowledging that, while our study provides potential mechanistic insights, temozolomide (TMZ) remains the current gold standard for GBM chemotherapy, more so than CDDP. The oral bioavailability and superior BBB penetration of TMZ position it as the preferred therapeutic agent in clinical practice [25]. However, resistance associated with TMZ remains a clinical challenge, given that most GBM patients eventually develop refractory disease [26]. Clinical trials have shown that CDDP is effective in recurrent GBM and can exert additive cytotoxic effects when combined with TMZ [27,28]. Accordingly, the findings with CDDP reported here provide added mechanistic insights into the added value of redox-targeted combination therapy, which is of value for future investigations with TMZ and other clinically relevant agents.

U87 and U87-EGFRvIII cells are established experimental models in GBM research with distinguishable biological and molecular profiles [29]. U87 cell line is the CDDP-sensitive GBM phenotype linked with moderate proliferation and resistance to oxidative stress and apoptosis. Previous studies showed that CDDP activates the EGFR signaling pathways, thereby reducing the sensitivity of malignant cells to CDDP [30–32]. In contrast, U87-EGFRvIII cells overexpress the constitutively active EGFR variant III (EGFRvIII) frequently associated with high-grade gliomas [33,34]. This mutation drives aggressive tumor behavior, such as increased cellular proliferation and invasiveness, and resistance to chemotherapy [29,35]. In addition, U87-EGFRvIII cells possess higher antioxidant capacity and altered redox balance, and thus reduced response to DNA-damaging agents, including CDDP [35]. This makes U87-EGFRvIII cells an ideal model for investigating drug resistance and evaluating redox-targeted GBM treatment [29,35]. To our knowledge, this is the first study investigating the combinatorial effects of C-PC and CDDP on GBM.

We evaluated the cytotoxic effect of C-PC on U87 and U87-EGFRvIII cells and HUVEC cells. Results confirmed that C-PC selectively reduces the viability of GBM cells, particularly the drug-resistant U87-EGFRvIII line, while sparing normal control cells, thus underscoring the safety profile of C-PC [18]. Compared to U87 cells, U87-EGFRvIII cells exhibited increased sensitivity due to their higher uptake of C-PC [29]. These findings align with reports demonstrating the selective cytotoxicity of C-PC against aggressive and drug-resistant cancer phenotypes [36], exemplified by the reported effectiveness of C-PC against the highly aggressive MDA-MB-231 breast cancer (BC) cell line when compared to other BC cell lines, while sparing normal cells [37].

Mechanistically, we tested the contribution of increased apoptosis as a mechanism underlying the cytotoxic effect of C-PC on U87 and U87-EGFRvIII cells. Our results showed that C-PC induces apoptotic cell death via caspase-3 activation and PARP cleavage, along with significant nuclear fragmentation in the aggressive U87-EGFRvIII cancer cells, but not in U87 cells. This aligns with previous studies showing that C-PC induces apoptosis via caspase activation in different cancer cell lines such as pancreatic, colorectal, breast, and liver cancer cell lines [38–41]. Combining C-PC with CDDP enhanced apoptosis in U87 cells, indicating an additive interaction, as also demonstrated for lung cancer [42]. On the other hand, the C-PC-CDDP combination did not improve cytotoxicity in U87-EGFRvIII cells, suggesting intrinsic EGFRvIII-mediated resistance mechanisms that limit CDDP efficacy in the presence of C-PC [43].

Changes in ROS are major contributors to the stages of cancer development, including tumor initiation, progression, invasion, metastasis, microenvironment remodeling, and therapeutic resistance [44,45]. Our results highlight the role of C-PC in regulating the redox balance, as evidenced by its capacity to significantly reduce the production of O_2_•, either alone or in combination with CDDP, and notably in U87-EGFRvIII cells. This was supported by the findings that elevated steady-state ROS levels in neoplastic cells make them more vulnerable to oxidative stress than normal cells [46], and that apoptotic cell death in malignant cells linked with conventional anti-cancer chemotherapy is exacted partly through inducing ROS production [47], noting side effects on normal tissues [48]. This indicated novel strategies based on antioxidants to target cancer cell death while sparing normal cells selectively [49]. In addition, that dihydromyricetin-induced ROS downregulation was demonstrated to activate caspase-9 and trigger PARP cleavage, leading to apoptotic cell death in human hepatoma HepG2 cells [50], and that the flavonoid dihydromyricetin reportedly induced apoptosis in Hepal-6 hepatoma cells by reducing ROS generation via the TGF-β/Smad3 pathway [51].

Insofar as antioxidants, such as MnSOD control ROS production [52], by catalyzing the dismutation of O_2_·^−^ into H_2_O_2_ [53], we report that the suppression of redox was linked by enhanced MnSOD expression and enhanced catalase activity, two key antioxidant enzymes known to regulate oxidative stress and apoptosis. Our findings indicate that C-PC increased MnSOD protein expression in U87-EGFRvIII cells. C-PC-induced increase in MnSOD was also associated with reduced levels of O_2_·^−^ and H_2_O_2_, leading to inhibited cell proliferation and apoptosis induction. MnSOD overexpression was documented to inhibit the proliferation of glioma cells [54,55], supporting the notion that antioxidant therapies are associated with increased expression of MnSOD and catalase [56], including SOD supplementation linked with upregulated caspase-3 [57], sensitize cells to oxidative stress-mediated apoptosis, and thus serve as potential anticancer strategies.

This mechanism linking lowered ROS levels with C-PC’s cytotoxicity toward cell death is not fully understood and was suggested to be exerted by activating the HIF-1α-SERPINE1 signaling pathway [58]. While the chemo-sensitization capacity of C-PC was reported for several cancer cell lines [16,21,43], its efficacy in the C-PC-CDDP combination remains largely unexplored. C-PC improved CDDP efficacy in U87 cells compared to CDDP alone but failed to affect cytotoxicity in U87-EGFRvIII cells, suggesting that C-PC as a standalone therapy is preferred for targeting resistant GBM subtypes. On the other hand, co-treatment with C-PC and CDDP enhanced caspase-3 activation and PARP cleavage, thereby accelerating apoptosis. The significant reduction of ROS production and increased MnSOD expression and catalase activity linked with C-PC-CDDP co-treatment of U87 cells point to redox homeostasis disruption as a mechanism of action [59].

These findings are consistent with prior reports showing that Spirulina-derived C-PC potentiated CDDP antitumor activity in Ehrlich ascites carcinoma-bearing mice [60] and that combining C-PC with topotecan increased cytotoxicity of LNCaP prostate cancer cells compared to topotecan alone, likely by augmenting caspase-3 and caspase-9 expression [61]. Similarly, a recent study reported that combined treatment of Micotherapy U-Care with CDDP increased SOD1 and SOD2 expression in GBM cells, leading to reduced oxidative stress and tumorigenesis [62].

On the other hand, the C-PC-CDDP combination did not improve efficacy compared to C-PC-treated U87-EGFRvIII cells. These findings indicate that the combination does not overcome CDDP resistance in U87-EGFRvIII cells, suggesting that C-PC may be more effective as a standalone therapy for resistant GBM subtypes [36,43].

While our findings on the C-PC’s redox-modulatory effects are promising, several limitations of our study should be acknowledged. First, while mechanistically informative, the use of CDDP does not accurately reflect current clinical practice, which indicates that TMZ constitutes the standard chemotherapy for GBM. This necessitates that future studies prioritize investigating C-PC-TMZ combinations for enhancing clinical relevance. Second, our *in vitro* findings require validation in clinically relevant models, including BBB-penetrant delivery systems, as well as *in vivo* models of GBM. Third, considering the bioavailability and BBB penetration properties, it is recommended that the concentrations of C-PC used in our study be optimized for clinical translation. Fourth, our study focused on two cell lines, thereby questioning the generalizability of the findings when tested across diverse GBM subtypes with different molecular characteristics.

## 5 Conclusion

This study provides mechanistic evidence for the dual therapeutic potential of C-PC in treating GBM. We confirm that C-PC selectively targets GBM cells by enhancing cellular uptake in resistant phenotypes while sparing normal cells, and that C-PC-CDDP combination therapy enhances cytotoxicity significantly in treatment-sensitive U87 cells through apoptosis induction and ROS modulation, the latter involving upregulated MnSOD and catalase expression, and C-PC monotherapy offers superior efficacy compared to combination treatment in CDDP-resistant U87-EGFRvIII cells. While these findings provide mechanistic insights, several limitations must be acknowledged for clinical translation. Studies investigating combinations of C-PC with temozolomide, the current gold standard for GBM treatment, and addressing the challenges of BBB penetration for both agents are warranted. In addition, *in vivo* validation and optimization of clinically relevant concentrations are essential subsequent steps. Despite these and other shortcomings, our work lays the groundwork for developing redox-targeted combination therapies that may ultimately enhance outcomes for GBM patients.

## Figures and Tables

**Figure A1:**
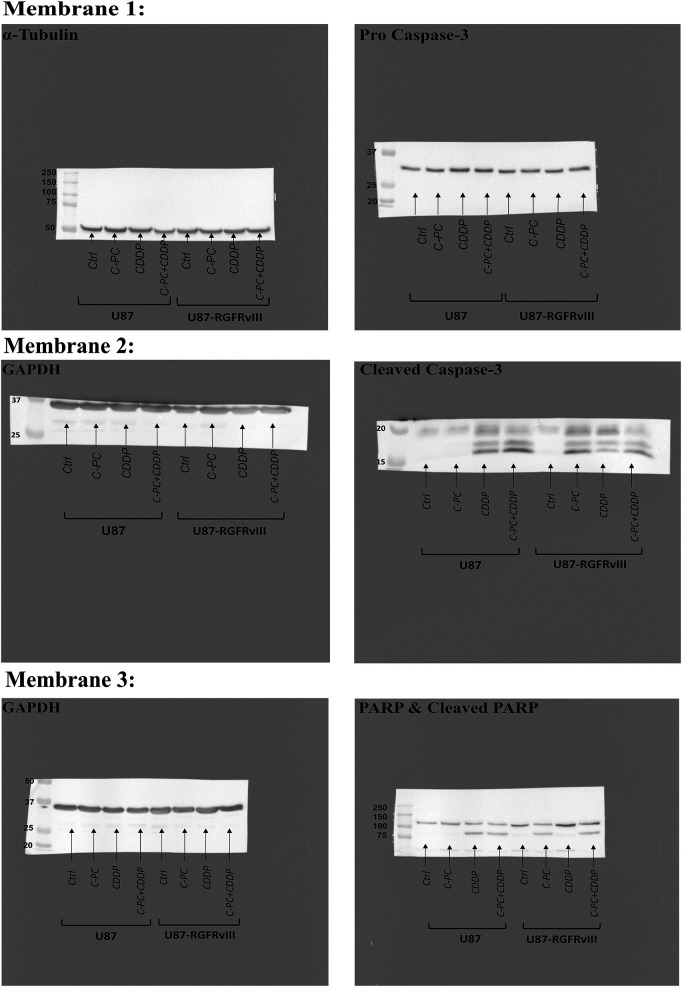
Original full-length Western blot corresponding to Fig. 5A

**Figure A2:**
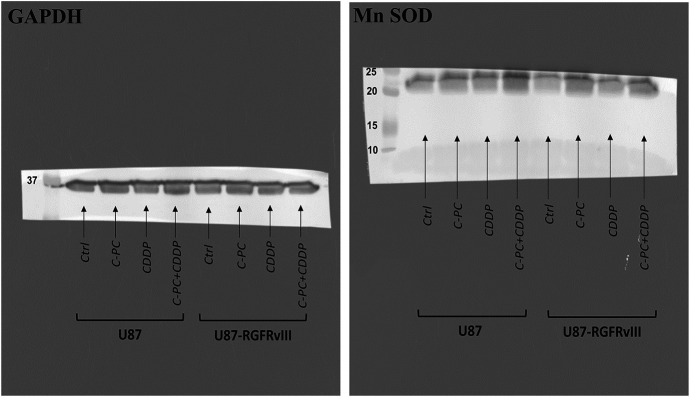
Original full-length Western blot corresponding to Fig. 7A

## Data Availability

The authors confirm that the data supporting the findings of this study are available within the article.
